# Genome-wide DNA methylation patterns associated with general psychopathology in children

**DOI:** 10.1016/j.jpsychires.2021.05.029

**Published:** 2021-08

**Authors:** Jolien Rijlaarsdam, Edward D. Barker, Chiara Caserini, M. Elisabeth Koopman-Verhoeff, Rosa H. Mulder, Janine F. Felix, Charlotte A.M. Cecil

**Affiliations:** a Department of Child and Adolescent Psychiatry/ Psychology, Erasmus MC University Medical Center Rotterdam, Rotterdam, the Netherlands; b Department of Psychology, Institute of Psychiatry, Psychology and Neuroscience, King's College London, UK; c Department of Psychology, Sigmund Freud University, Milan, Italy; d The Generation R Study Group, Erasmus MC University Medical Center Rotterdam, Rotterdam, the Netherlands; e Department of Pediatrics, Erasmus MC University Medical Center Rotterdam, Rotterdam, the Netherlands; f Department of Epidemiology, Erasmus MC University Medical Center Rotterdam, Rotterdam, the Netherlands; g Molecular Epidemiology, Department of Biomedical Data Sciences, Leiden University Medical Center, Leiden, the Netherlands

**Keywords:** DNA methylation, General psychopathology, Epigenetics, Generation R, ALSPAC

## Abstract

Psychiatric symptoms are interrelated and found to be largely captured by a general psychopathology factor (GPF). Although epigenetic mechanisms, such as DNA methylation (DNAm), have been linked to individual psychiatric outcomes, associations with GPF remain unclear. Using data from 440 children aged 10 years participating in the Generation R Study, we examined the associations of DNAm with both general and specific (internalizing, externalizing) factors of psychopathology. Genome-wide DNAm levels, measured in peripheral blood using the Illumina 450K array, were clustered into wider co-methylation networks (‘modules’) using a weighted gene co-expression network analysis. One co-methylated module associated with GPF after multiple testing correction, while none associated with the specific factors. This module comprised of 218 CpG probes, of which 198 mapped onto different genes. The CpG most strongly driving the association with GPF was annotated to FZD1, a gene that has been implicated in schizophrenia and wider neurological processes. Associations between the probes contained in the co-methylated module and GPF were supported in an independent sample of children from the Avon Longitudinal Study of Parents and Children (ALSPAC), as evidenced by significant correlations in effect sizes. These findings might contribute to improving our understanding of dynamic molecular processes underlying complex psychiatric phenotypes.

## Highlights

• This study identified genome-wide DNAm patterns that associated to the general factor of psychopathology after multiple testing correction.
• Symptom-specific internalizing and externalizing factors were unrelated to DNAm patterns.
• The CpG most strongly driving this association was annotated to *FZD1*, a gene that has been implicated in neurological processes.
• Associations were supported in an independent cohort, as evidenced by significant correlations in effect sizes.

## 1 Introduction

Psychiatric disorders and their symptoms co-occur more often than expected by chance (Angold et al., 1999; Kessler et al., 2005) and show continuity across development (Wichstrom et al., 2017). Although psychiatric symptoms typically cluster in broadly defined internalizing and externalizing domains, those domains are in themselves correlated as well (Achenbach et al., 2016). There is growing evidence that the shared versus specific variance between psychiatric disorders and their symptoms may be usefully represented by a general psychopathology factor (GPF) (for studies in childhood and early adolescence, see: Lahey et al., 2015; Neumann et al., 2016; Olino et al., 2014; Patalay et al., 2015; Rijlaarsdam et al., 2021). According to recent research, GPF shows structural temporal stability in childhood (Rijlaarsdam et al., 2021) throughout adulthood (Gluschkoff et al., 2019). Furthermore, GPF in childhood has been found to predict the course and severity of a multitude of psychiatric outcomes in adolescence, over and above specific psychopathology dimensions (Pettersson et al., 2018).

Previous research found evidence for both genetic and environmental influences on GPF. For example, GPF has demonstrated a significant single nucleotide polymorphism (SNP) heritability of 38% (Neumann et al., 2016). Furthermore, it has been repeatedly shown that common genetic risk variants associated with childhood psychiatric outcomes, as indexed by polygenic risk scores (PRS), underlie GPF (Brikell et al., 2018; Riglin et al., 2019). This is supported by studies showing that genetic influences are often pleiotropic, simultaneously affecting multiple psychiatric outcomes.(Cross-Disorder Group of the Psychiatric Genomics Consortium, 2013; Cross-Disorder Group of the Psychiatric Genomics Consortium, 2019) Similarly, environmental factors such as childhood maltreatment have been associated with a wide range of psychiatric outcomes, ranging from anxiety and depression to rule-breaking and aggression (Vachon et al., 2015). Interestingly, childhood maltreatment was found to associate most strongly to GPF as opposed to symptom-specific factors (e.g., internalizing, externalizing) (Brodbeck et al., 2018; Caspi et al., 2014). These genetic and environmental factors are known to interact, for example with strongest effects of maltreatment on mental health shown for those children at high genetic risk (Jaffee et al., 2005).

However, the molecular mechanisms underlying these genetic and environmental influences on GPF remain unclear. Epigenetic mechanisms that regulate gene expression, such as DNA methylation (DNAm), have emerged as promising candidates. Studies have shown that DNAm (i) responds to both genetic and environmental factors (Meaney, 2010) and (ii) associates with psychiatric outcomes across the genome (Cecil et al., 2018; Neumann et al., 2020; Walton et al., 2017). As such, DNAm may represent a potential molecular mechanism that could explain psychiatric disease susceptibility across the life span. However, while shown to associate to separate psychiatric outcomes (e.g., attention-deficit/hyperactivity disorder symptoms, conduct problems), DNAm has not been examined in relation to GPF. Consequently, it is not known yet whether the identified associations are specific to these psychiatric outcomes or shared between them.

Using data from 440 children aged 10 years participating in the Generation R Study, a population-based sample, we examined the associations of genome-wide DNAm with both general and specific factors of psychopathology. Given how little we know of the underlying biology of general psychopathology, our aim was to investigate epigenetic correlates to help identify targets for future research. Specifically, identified targets could be followed up in future to establish the potential utility of DNAm as a non-causal biomarker versus a potential mechanistic underpinning of general psychopathology. Because DNAm levels have been shown to correlate substantially between CpG sites (Kuan and Chiang, 2012), we examined wider biological networks (so called ‘modules’) of co-methylated DNAm sites. Modules that significantly associated with general psychopathology were followed-up using bioinformatics tools and online databases to characterize (i) CpG sites in the module driving the associations, (ii) enriched biological pathways, (iii) potential genetic influences (e.g., methylation quantitative trait loci [mQTLs]), (iv) cross-tissue correspondence of DNAm levels, and (v) the relationship with gene expression in peripheral and neural tissue. We further examined major life events from birth to age 10, including maltreatment, as a potential environmental factor that may influence DNAm levels of any module associated with GPF. The replicability of our findings was tested in an independent cohort, the Avon Longitudinal Study of Parents and Children (ALSPAC).

## 2 Methods

### 2.1 Participants

This study is embedded in the Generation R Study, an ongoing population-based study of children born to pregnant women residing in Rotterdam, the Netherlands, with expected delivery dates between April 2002 and January 2006 (Kooijman et al., 2016). For this cross-sectional research, we included 10-year-old children of European ancestry who had available data on DNA methylation (DNAm) and general psychopathology (N = 440, 50.7% male). Written informed consent was obtained from all parents. The study was conducted in accordance with the guidelines proposed in the World Medical Association Declaration of Helsinki and was approved by the Medical Ethical Committee of the Erasmus MC, University Medical Center, Rotterdam.

### 2.2 Measures

#### 2.2.1 DNA methylation

Five-hundred nanograms of genomic DNA from blood was bisulfite converted using the EZ-96 DNA Methylation kit (Shallow) (Zymo Research Corporation, Irvine, USA). Samples were plated onto 96-well plates in no specific order. DNAm was quantified using the Illumina Infinium HumanMethylation450 BeadChip (Illumina Inc., San Diego, USA). For further information on the preparation and normalization of the HumanMethylation450 BeadChip array, see Supplementary Information 1.

In this study we examined networks of co-methylated DNAm sites that have been previously extracted and characterized in Generation R based on all children with DNAm data at age 10 (Koopman-Verhoeff et al., 2020), using a weighted gene co-expression network analysis in R (WGCNA package; Langfelder and Horvath, 2008). Utilizing the correlation patterns between the individual sites, WGCNA is a system-level data reduction approach that reduces the dimensionality of the data and may provide insight into wider DNAm networks (so called ‘modules’) associated with a phenotype of interest. Block-wise network construction was run using default settings (power threshold of 6; minimal module size of 30 sites; merge cut height of 0.25). Each derived module was colored by size automatically and summarized by a ‘module eigengene’ (ME) value, the first principal component of the given module. CpG sites that did not co-methylate were assigned to an ‘unclassified’ module. We ordered and numbered the modules according to their strength of association with GPF for simplicity. WGCNA identified 64 co-methylated modules, containing between 30 and 65,804 CpG sites. The majority of sites (*n* = 261,374) were unclassified, suggesting that their inter-correlations were too low to constitute modules.

#### 2.2.2 General psychopathology

Psychopathology symptoms were assessed with the Child Behavior Checklist 6–18 (CBCL/6–18; Achenbach and Rescorla, 2001), a validated and widely used parental assessment of a child's behavioral and emotional problems. Mothers completed questions about a range of emotional and behavioral problems of the child in the past six months on a three-point scale (0 = not true, 1 = somewhat true, 2 = very true). See statistical analysis section for details on the GPF model.

#### 2.2.3 Covariates

All analyses were adjusted for sex, sample plate, and estimates of cell type proportions (Houseman et al., 2012). In sensitivity analyses, we additionally controlled for a range of potential factors that may influence general psychopathology or have been typically adjusted for in birth cohorts (Dunn et al., 2020). These extended covariates included: age at the assessment of general psychopathology (mean = 9.69 ± 0.25), maternal smoking during pregnancy (never smoked in pregnancy [78.8%]; smoked until pregnancy was known [10.0%]; continued smoking [11.2%]), maternal age at intake (mean = 32.19 ± 4.00), maternal educational level (higher vocational education and university: yes [67.3%] or no [32.7%]), marital status (married or cohabiting [95.4%] versus single [4.6%]), and maternal psychopathology symptoms (mean = 0.15 ± 0.19) measured at 20 weeks of pregnancy, using the Global Severity Index derived from the Brief Symptom Inventory, a self-report mental health symptom scale (Derogatis, 1993).

#### 2.2.4 Major life events

To investigate major life events as a potential environmental factor that may influence DNAm levels of any module associated with GPF, we used a major life events inventory (Amone-P'Olak et al., 2009; Brown and Harris, 1978) encompassing the intervening years from birth to age 10. Mothers were asked to indicate whether their 10-year-old child had ever experienced specific life events (e.g., exposure to physical or sexual violence, serious illness or dead in the family, family economic pressure or conflict).

### 2.3 Statistical analysis

Statistical analyses were performed in R (R Core Team, 2017). GPF scores were winsorized at ±3 SD and standardized. The analyses proceeded in three main steps.

#### 2.3.1 Step 1: general psychopathology factor

In the first step, we used confirmatory factor analysis (CFA) to fit a hierarchical general psychopathology model with the Lavaan statistical package (Rosseel, 2012) in the full sample with CBCL data available (*N* = 4954). Whereas a single general factor loaded on all CBCL problem subscales, two specific factors loaded on internalizing (anxious/depressed, withdrawn/depressed, somatic complaints) versus externalizing (rule-breaking behavior, aggressive behavior, attention problems) subscales. Three subscales (social problems, thought problems, other problems) were indicators of the general factor but not of the specific factors because the CBCL did not group them into any higher order domain. The internalizing and externalizing factors were allowed to correlate with each other but not with the general factor. Consequently, the general factor represents a common vulnerability across a broad range of both internalizing and externalizing problems (i.e., explaining the shared variance among all problem scales) that is independent of the more specific internalizing and externalizing factors.

We performed a correlated two-factors model for further validation (see Supplementary Figure 1). Specifically, internalizing and externalizing factors were again (i) each indicated by a subset of psychopathology domains and (ii) assumed to be correlated. However, no general factor was modeled. Model fit was established using the robust comparative fit index (CFI; acceptable fit ≥ 0.90), root-mean-square error of approximation (RMSEA: acceptable fit ≤ 0.08), standardized root mean square residual (SRMR: acceptable fit ≤ 0.08) and the bayesian information criterion (BIC; lower levels indicating better fit) (Hu and Bentler, 1999; Raftery, 1995).

#### 2.3.2 Step 2: associations between DNA methylation and general psychopathology

Second, we regressed the co-methylated modules on the general and specific psychopathology factor scores correcting for sex, sample plate, and estimates of cell type proportions. Associations were considered significant if they survived Bonferroni correction for multiple testing [corrected *p*-value = 0.05/(64 modules*3 outcomes) = 0.0002604]. In sensitivity analyses, we reran associations for significant co-methylated modules, additionally adjusting for extended covariates. Missing values of these extended covariates (range = 0.3–8.2%) were imputed twenty times and results were pooled using the MICE software package (van Buuren and Groothuis-Oudshoorn, 2011).

Modules that significantly associated with general psychopathology were then followed-up to characterize (i) the individual CpG probes in the module driving the associations, (ii) enriched biological pathways, (iii) potential genetic and environmental influences, (iv) cross-tissue correspondence, and (v) the relationship with gene expression. First, the individual CpG probes contained in the modules were regressed on GPF using the CpGassoc package (Barfield, Kilaru, Smith and Conneely, 2012), correcting for sex, sample plate, and estimates of cell type proportions. Second, in order to identify enriched biological pathways within a module, we used the gene ontology (GO) method from the missMethyl R package including genes mapped to CpGs contained in the module (Phipson et al., 2016). GO terms were considered significant at a false discovery rate (FDR<0.05). Third, we explored potential genetic and environmental influences on DNAm. We first used a heritability tool characterizing additive genetic influences as opposed to shared and non-shared environmental influences on DNAm, based on data from monozygotic and dizygotic twins (Hannon et al., 2018). We then used the methylation quantitative trait loci database resource (mQTLdb, GCTA set, childhood; Gaunt et al., 2016) as a more specific tool for identifying genetic influences on DNAm levels in childhood. With regards to potential environmental influences, we examined whether DNAm levels of any module associated with GPF were also associated with major life events. Fourth, we characterized cross-tissue correspondence of DNAm using the blood-brain concordance tool (Hannon et al., 2015) based on postmortem data from 122 individuals with DNAm from whole blood and four brain regions. Finally, for genes associated with CpGs contained in significant modules, tissue expression was investigated using the Genotype-Tissue Expression (GTEx) data resource (GTEx Consortium, 2015).

#### 2.3.3 Step 3: replication

In the third step, we used data from the accessible resource for integrated epigenomics studies (ARIES; Relton et al., 2015) (www.ariesepigenomics.org.uk) to test the replicability of our findings. ARIES is nested within the Avon Longitudinal Study of Parents and Children (ALSPAC), an ongoing epidemiological study of children born to 14,541 pregnant women residing in Avon, United Kingdom, with an expected delivery date between April 1991 and December 1992 (85% of eligible population; Fraser et al., 2013). Ethical approval for the study was obtained from the ALSPAC Ethics and Law Committee and the Local Research Ethics Committees. Informed consent was obtained from all ALSPAC participants. The original ALSPAC sample is representative of the general population (Boyd et al., 2013). Please note that the study website contains details of all the data that is available through a fully searchable data dictionary and variable search tool: http://www.bristol.ac.uk/alspac/researchers/our-data/. For this study, we included children from ARIES who had DNAm data at age 7 measured using the Illumina 450K Infinium BeadChip, as well as data on general psychopathology at age 7 (*N* = 811, 50.0% male). See supplementary Information 1 and 2 for further details on the DNAm data and the general psychopathology factor in ALSPAC.

For each co-methylated module associated with general psychopathology in Generation R, probes were extracted in ALSPAC and individually regressed on the general psychopathology factor, correcting for sex, sample plate, and estimates of cell type proportions. To examine convergence between the Generation R and ALSPAC samples, the Pearson correlation of the effect sizes between the samples was calculated. We also considered the sign of the regression coefficients.

## 3 Results

### 3.1 Step 1: general psychopathology factor

As displayed in Supplementary Information 2, both the hierarchical (general psychopathology, internalizing, externalizing factors) model (CFI = 0.963; RMSEA = 0.088, SRMSR = 0.030; BIC = 178722.864) and the correlated two-factors (internalizing, externalizing factors) model (CFI = 0.984; RMSEA = 0.063; SRMR = 0.023, BIC = 123497.825) showed acceptable model fit. All factor loadings of the internalizing and externalizing factors decreased substantially from the correlated-factors model to the hierarchical model. Specifically, whereas all factor loadings of GPF were well above 0.30 (average loading = 0,69, range = 0.46–0.81), this was not the case for the specific internalizing (average loading = 0.31, range = 0.20–0.47) and externalizing (average loading = 0.29, range 0.09–0.48) factors. In the hierarchical model, the specific internalizing and externalizing factors correlated negatively with each other. This is to be expected, as the shared variance is already captured by GPF.

In light of the relatively low factor loadings for the specific factors versus GPF in the hierarchical model, GPF will be of primary focus in all subsequent analyses. Furthermore, in sensitivity analyses, we additionally assessed the internalizing and externalizing factor scores from the correlated two-factors model in relation to DNAm.

### 3.2 Step 2: associations between DNA methylation and general psychopathology

As shown in Table 1, one co-methylated module (module 1) was significantly associated with GPF after Bonferroni correction, *b* = 0.0047 (95%CI = 0.0022–0.0072), β = 0.10, *p* = 2.33*10–4. Effect estimates remained largely unchanged after adjusting for the extended covariates in a sensitivity analysis, *b* = 0.0050, *p* = 1.81*10–4. No modules were associated with the specific internalizing and externalizing factors from the hierarchical and the correlated two-factors models after Bonferroni correction. Supplementary Table 1 shows the full linear regression results.

**Table 1:** Associations between DNA methylation patterns and child psychopathology.

	General psychopathology factor				Internalizing factor				Externalizing factor		
Module	P-value	*b*	SE		P-value	*b*	SE		P-value	*b*	SE
1	2.33E-04	0.00473	0.00128		0.026212	0.00292	0.00131		0.343037	−0.00124	0.00131

Module 1 comprised of 218 CpG probes, of which 198 mapped onto different genes located on all 22 autosomal chromosomes. As shown in Supplementary Table 2, DNAm levels at the CpG probes within module 1 were moderately to highly correlated (mean *r* = 0.42). Linear regression showed that 80 (37%) of the 218 individual CpG probes contained in module 1 were associated with GPF at a nominal *p*-value threshold of 0.05 (see Supplementary Table 3, sorted by *p*-value). The top 10 individual CpG probes in module 1 associated with GPF are displayed in Table 2. Twenty-seven of the CpG probes included in the module were previously identified as polymorphic (i.e., overlapping with SNPs) (Chen et al., 2013). These 27 CpGs included two of the top 10 CpGs driving the association with GPF. GO analysis revealed no significantly enriched common biological processes, cellular components, or molecular functions for the genes mapped to the individual CpGs contained in module 1 (see Supplementary Table 4).

**Table 2:** Top 10 CpGs in Module 1 associated with the general factor of psychopathology.

CpG	P-value	*b*	*SE*	Position	Chromosome	Gene	Genomic location	Proximity to CpG island
cg13496324	4.78E-05	0.00237	0.00058	90893736	7	*FZD1*	TSS200	Island
cg16384885	5.65E-05	0.00178	0.00044	113930767	11	*ZBTB16*	TSS1500;5'UTR	Island
cg14756353	6.56E-05	0.00220	0.00054	121468984	3	*GOLGB1*	TSS1500	S_Shore
cg06671139	0.000190	0.00110	0.00029	73075935	15	*ADPGK*	1stExon;Body	Island
cg00563892	0.000191	0.00182	0.00048	42588392	2	*COX7A2L*	TSS200	Island
cg12570756	0.000221	0.00269	0.00072	809538	11	*RPLP2*	TSS1500	Island
cg00736283	0.000230	0.00204	0.00055	14247601	19	*ASF1B*	TSS200	Island
cg03925157	0.000257	0.00218	0.00059	17549955	20	*DSTN*	TSS1500	Island
cg00998813	0.000263	0.00237	0.00064	49894005	3	*TRAIP*	TSS200	Island
cg05951573	0.000269	0.00227	0.00062	150812282	7	*AGAP3*	Body	

A total of 214 (98%) probes in module 1 had twin heritability estimates available, which showed low additive genetic influences (mean *r* = 0.04, range = 4.40 E^−18^-0.37) versus moderate to strong shared (mean *r* = 0.60, range = 0.13–0.96) and non-shared (mean *r* = 0.36, range = 0.02–0.86) environmental influences (see Supplementary Table 5), suggesting that variation in these CpG probes may be primarily explained by environmental influences. This finding was supported by the mQTL tool, which showed that only two of the 218 probes in module 1 were associated with known mQTLs in childhood (see Supplementary Table 5). However, the cumulative score of exposure to major life events (*n* = 414, mean = 3.91, *SD* = 2.45, range 0–14) was unrelated to the co-methylated module as a whole, *b* = 0.0003 (95%CI = −0.0008–0.0014), β = 0.01, *p* = 0.635149. Regarding cross-tissue correspondence, we found that DNAm levels in blood correlated significantly with DNAm levels in at least one brain region for 116 (53.2%) of the 218 probes in module 1 (see Supplementary Table 6). Based on GTEx data, the genes mapped to the top 10 CpGs driving the association with GPF showed mixed profiles of expression in peripheral and neural tissue (see Supplementary Figure 2). Whereas two genes were specifically expressed in peripheral tissue groups, other genes, particularly *RPLP2* and *DSTN*, were more broadly expressed. However, none of the genes showed specific expression in the brain.

### 3.3 Step 3: replication

As a final step, we tested the replicability of our findings in ALSPAC, focusing on corresponding probes after quality control (*n*_module 1_ = 209). In ALSPAC, intercorrelations among the probes within module 1 were moderate to high (mean *r* = 0.60, see Supplementary Table 7). Although only 8 (3.8%) of the 209 probes were nominally significantly associated with GPF in ALSPAC, we were interested mainly in general convergence between effect estimates at the network level. That is, the overall concordance in effect sizes between Generation R and ALSPAC (see Supplementary Table 8) was significant, *r* (209) = 0.31, *p* < 0.001, and the direction of associations was mostly (79.9%) consistent across the two cohorts. Furthermore, the concordance in effect sizes between the cohorts was stronger when restricting to the module 1 probes that were nominally associated with the general psychopathology factor in Generation R, *r* (79) = 0.44, *p* < 0.001.

## 4 Discussion

This study used a network-based approach to examine associations of genome-wide DNAm with both general and specific factors of psychopathology in a population-based sample of children aged 10 years. We highlight here three key findings. First, we identified one co-methylated module associated with GPF after multiple testing correction, but we did not observe associations with the specific internalizing and externalizing factors. Second, functional characterization of the sites contained in this module suggested that variation may be best explained by environmental as opposed to genetic influences. Third, associations were supported in an independent sample, as evidenced by significant correlations in effect sizes.

Out of a total of 64 co-methylated modules identified across the genome, one was found to associate with the general psychopathology factor, after multiple testing correction. This module comprised of 218 CpGs, 80 (37%) of which nominally associated with the general psychopathology factor. The CpG most strongly driving this association was annotated to *FZD1*, a gene involved in the Wnt signaling pathway that (i) is essential for neurological development and the maintenance of the nervous system, and (ii) has been associated with susceptibility to neurological disorders (Okerlund and Cheyette, 2011; Patapoutian and Reichardt, 2000). In a recent case-control genome-wide association study (GWAS) of schizophrenia (Liu et al., 2020), four of the top five signals were located in *FZD1*. This finding was supported by post-GWAS gene-based analyses, showing that multiple markers in *FZD1* are involved in the susceptibility to schizophrenia. Whereas GTEx data suggests that *FZD1* is not specifically expressed in the brain but in peripheral tissues (e.g., thyroid, tibial nerve and artery), Pietersen et al. (2014) found *FZD1* to be upregulated in the superior temporal cortex in postmortem schizophrenia brain. Interestingly, in adults, it has been found that a thought disorder factor, as indicated by schizophrenia, mania, and obsessive-compulsive disorder, was almost perfectly correlated with GPF, suggesting that it may represent its core feature (Caspi et al., 2014). Also of interest, the second most strongly associated CpG in the co-methylated module was annotated to *ZBTB16*, a protein coding gene involved in the cell cycle progression that has been linked to various psychiatric outcomes bridging the internalizing and externalizing divide, such as conduct disorder symptoms (Sonuga-Barke et al., 2008), ADHD (Zayats et al., 2015), and major depressive disorder (Spijker et al., 2010).

Of note, the 218 CpGs contained in the co-methylated module were not restricted to a specific genomic location, but spanned different genes (*n* = 198) and all 22 autosomal chromosomes. This finding supports previous research showing scattered genomic findings for differentially methylated probes associated with an increase in psychopathology symptoms (low-high CBCL total scores) in youths at a 3-year follow-up (Spindola et al., 2019). Future studies are needed to identify any potential common characteristic of these CpG sites. For example, transcription factors might bind to regulatory sites affecting the expression levels of multiple genes, potentially influencing a common vulnerability to psychopathology (Tong et al., 2017). Using publicly available tools (i.e., twin-based heritability tool, mQTLdb), we found little evidence of genetic influences on DNAm levels of the CpGs contained in the co-methylated module within childhood. Although it seems that these sites are mostly influenced by environmental as opposed to genetic factors, the association between the module and the general psychopathology factor was robust to adjustment for factors that are typically controlled for in birth cohorts (e.g., maternal education and psychopathology). Furthermore, a cumulative score of major life events (including physical and sexual abuse) encompassing the intervening years from birth to age 10 was unrelated to the co-methylated module. As such, it is unclear what factors influence methylomic variation within this module, and future studies will be needed to identify whether specific adversities not included in the present study might be at play.

We found some support for the associations between the CpGs contained in the co-methylated module and GPF in ALSPAC, an independent population based cohort. While only few of the individual CpGs contained in the co-methylated module nominally associated with GPF in ALSPAC, we observed a wider concordance in effect sizes and the direction of associations across CpG sites included in the module. Although this finding supports our use of clusters of highly co-methylated DNAm sites across the genome, several study differences should be noted. First, the children in the Generation R Study were on average 10 years of age, whereas the ALSPAC sample included 7-year-olds. Second, the GPF was fitted to dimensional (CBCL, questionnaire) versus more clinically oriented categorical (DAWBA, interview) data in Generation R and ALSPAC, respectively. Any of these study differences may account for disparity in results.

The present findings should be interpreted in the light of several limitations. First, given that the current study was cross-sectional in nature, the direction of the association between DNAm and the GPF cannot be inferred. Second, by identifying clusters of highly co-methylated DNAm sites across the genome, our analysis was performed over wider DNAm networks, and not at the individual CpG-level. It remains to be determined, however, whether these networks are functionally relevant. In particular, integration of transcriptomic data will be important for assessing the functional relevance of DNAm changes to gene expression. Third, because DNAm is tissue specific, observations in peripheral blood may not reflect other tissues of interest (e.g., the brain) that are unavailable for population-based studies of living individuals. Fourth, although it is interesting that no associations were observed for the specific internalizing and externalizing factors, these specific factors also showed the lowest loadings. However, we demonstrated that despite acceptable factor loadings, the internalizing and externalizing factors from the correlated two-factors model (where no general factor was modeled) were also unrelated to DNAm.

In summary, the current study identified genome-wide DNAm patterns that associated to GPF as opposed to specific internalizing and externalizing factors. Showing some support for this link between DNAm and GPF in an independent sample of children, findings of this study might contribute to improving our understanding of dynamic molecular processes underlying complex psychiatric phenotypes.

## Declaration of competing interest

None.

## Author statement

Data collection was performed by the Generation R and ALSPAC teams. JR participated in study design, literature review, data analysis, interpretation of the findings, and writing. CAMC participated in study design, interpretation of the findings, and writing. CC conducted statistical analyses and contributed to the literature review. EDB, JFF, RHM and MEK contributed to resources and critically reviewed the manuscript.
